# Improvements in Smartphone and Night Vision Imaging Technologies Enable Low Cost, On-Site Assays of Bioluminescent Cells

**DOI:** 10.3389/fbioe.2021.767313

**Published:** 2021-11-19

**Authors:** Mark Wienhold, Andrew Kirkpatrick, Tingting Xu, Steven Ripp, Gary Sayler, Dan Close

**Affiliations:** ^1^ 490 BioTech, Inc., Knoxville, TN, United States; ^2^ Center for Environmental Biotechnology, The University of Tennessee, Knoxville, TN, United States

**Keywords:** bioluminescence, luciferase, optical imaging, remote sensing, smartphone, portable analytical device, toxicity

## Abstract

Technologies enabling on-site environmental detection or medical diagnostics in resource-limited settings have a strong disruptive potential compared to current analytical approaches that require trained personnel in laboratories with immobile, resource intensive instrumentation. Handheld devices, such as smartphones, are now routinely produced with CPUs, RAM, wireless data transfer capabilities, and high-resolution complementary metal oxide semiconductor (CMOS) cameras capable of supporting the capture and processing of bioluminescent signals. In theory, combining the capabilities of these devices with continuously bioluminescent human cell-based bioreporters would allow them to replicate the functionality of more expensive, more complex, and less flexible platforms while supporting human-relevant conclusions. In this work, we compare the performance of smartphone (CMOS) and night vision (image intensifier) devices with *in vivo* (CCD camera), and *in vitro* (photomultiplier tube) laboratory instrumentation for monitoring signal dynamics from continuously bioluminescent human cellular models under toxic, stable, and induced expression scenarios. All systems detected bioluminescence from cells at common plating densities. While the *in vivo* and *in vitro* systems were more sensitive and detected signal dynamics representing cellular health changes earlier, the night vision and smartphone systems also detected these changes with relatively similar coefficients of variation and linear detection capabilities. The smartphone system did not detect transcriptional induction. The night vision system did detect transcriptional activation, but was less sensitive than the *in vivo* or *in vitro* systems and required a stronger induction before the change could be resolved.

## Introduction

Most laboratory grade fluorescent or luminescent screening instrumentation utilizes either charge coupled device (CCD) cameras or photomultiplier tubes (PMTs) as sensors. CCD cameras consist of many light-sensitive areas that convert photons into electrons when struck, such that the number of electrons collected will be directly proportional to the photon intensity recorded within each area. This allows them to be very sensitive, but also makes them vulnerable to dark current noise if not integrated with cooling systems to reduce thermal noise (Janesick, 2001). For laboratory use, they are often mounted atop a light-tight box where samples can be placed, but as their technology improves, they are starting to become imbedded within plate reader instrumentation to enable multimodal high-content imaging. PMTs are most commonly incorporated into plate reader-based instrumentation. Like CCD cameras they also report photon detection via the production of an electron. However, as their name suggests, that signal is then amplified using a series of electrodes to generate an output current proportional to the input flux. While this amplification procedure makes them highly sensitive, their incorporation into plate readers with integrated mechanical systems that move them from well to well, or move the plate relative to their position, results in devices that are relatively large and fragile and requires stable, higher voltage power supplies (Wright, 2017). The complexity and sensitivity of these types of sensors requires them to be integrated into instrumentation that can protect them from physical harm, supply them with the necessary power for optimal performance, and protect them from background light exposure. This often prevents their use in mobile operations, where small sizes, low power requirements, and robustness against environmental exposure is prioritized. Because of these reasons, there is an increased interest in the use of smaller, less expensive, and more easily obtainable instrumentation to perform biological assays, especially under low resource constraints.

Two optical detection devices readily available to the public and specifically designed for use under adverse conditions are night vision optics and smartphones. Originally produced for military applications, but now available to sportsman and for general use, night vision optics are often manufactured specifically to survive mobile use under harsh environmental conditions with minimal power requirements. Their sensor elements are image intensifiers that operate similar to PMTs. Within these sensors, photons strike a photocathode, are converted to electrons, and are then accelerated towards a higher voltage microchannel plate where they are amplified and retransmitted in a straight line towards a phosphor screen. Following absorption by the phosphor screen, they are reemitted as photons so they can be viewed or recorded by the observer (Haque and Muntjir, 2017). Although sparsely reported in biological research, night vision optics have previously been used to visualize luminescence, but not to quantify luminescent output.

Due to their ubiquity in modern society, smartphones are designed to meet similar usage requirements. The cameras in these devices are primarily based on complementary metal oxide semiconductor (CMOS) sensors. These sensors are similar to CCD sensors, but due to their method of manufacture can integrate a number of processing and control functions directly onto the sensor. Unlike CCDs, this allows them to operate with lower power consumption, a single master clock, and a single-voltage power supply (El Gamal and Eltoukhy, 2005). Although their performance was originally overshadowed by CCDs, due to competition in the consumer mobile electronics market and their prevalence in these devices, there has been heavy investment towards improving their performance that now makes them direct competitors with CCD sensors. CMOS sensors have broader representation in biological publications, having been used to measure chemiluminescence (Zangheri et al., 2015; Zangheri et al., 2021), and firefly (Kim et al., 2017; Michelini et al., 2019; Hattori et al., 2020) and bacterial luciferase (Ma et al., 2020) activity in animals, cultured human cells, and bacteria.

Bioluminescence has become an attractive assay modality for these applications because most study targets do not naturally produce luminescent signals, it can be easily measured using a variety of different sensor types, and host cells can modulate post-treatment signal intensity to achieve a wide detection range. One of the most common bioluminescent assay types is toxicity screening. It is well suited to this role because it can be genetically encoded into a target, such as a cell or bacterium, and the presence, absence, or change in intensity of the post-exposure bioluminescent signal provides an easily interpreted indication of the level of toxicity to the host. An example of this is the Microtox toxicity test, which is based on the use of a bioluminescent bacterium and has served as an official standard for acute toxicity screening in countries such as Germany (DIN 38412-26:1994-05) and the United States (ASTM method D5660-96). As our understanding of the complexities and dynamics of cytotoxic responses between species and within the different tissue types of a single species has improved (Slater, 2001), there has been a push to identify human-relevant toxicity using new assay procedures that can more quickly and inexpensively screen large numbers of samples, work within a variety of tissue types, and perform under a variety of conditions (Judson et al., 2013). Most of this testing relies on laboratory-based assays that require expensive, complex, and resource intensive analytical instrumentation to report the activation of specific cytotoxic pathways, the production of cytotoxic marker compounds such as released enzymes or reactive oxygen species, or the state of cellular metabolism as a representative proxy for cellular health (Fan and Wood, 2007). However, there is a growing body of work that seeks to perform these assays with lower cost, more readily available instrumentation in the hope of enabling more efficient testing or making testing available in areas without access to scientific facilities.

An additional complication for bioluminescent assays is that they are hindered by the high cost of the requisite chemical substrate addition that must be performed prior to each generation of signal, the hands-on time required to scale cultures due to obligatory sample destruction concurrent with interrogation, and the inability to provide continuously bioluminescent signals at timescales enabling detection by the less sensitive sensor technologies that are found in portable, inexpensive devices. A possible solution for overcoming these limitations is to replace their use with newer continuously bioluminescent reporter technologies that continuously produce bioluminescence and autonomously adjust signal intensity to reflect real-time changes in host viability (Xu et al., 2014; Conway et al., 2020). These reporter systems use the host’s rapidly fluctuating pool of FMNH_2_ to modulate signal intensity similar to how luciferin-dependent reporters use total ATP availability as a limiting reagent to control signal intensity. However, unlike externally excited reporters, they genetically encode both the luciferin and luciferase production components of the bioluminescent pathway. This allows them to continuously recycle luciferin, which negates the need to externally induce signal activation, potentiates bioluminescent signal production across the full host lifetime, and allows the phenotype to be passaged to further generations (Close et al., 2010).

Their ability to encode both the luciferase and luciferin synthesis components of the bioluminescent pathway enable them to function continuously, while their ability to assemble luciferin using only metabolites endogenous to the eukaryotic cytoplasm and their dependence on reducing power, rather than ATP, as a limiting reagent allows them to self-modulate signal intensity (Close et al., 2009). Comparative testing has shown that continuously bioluminescent technologies yield similar results to their luciferin-dependent counterparts (Class et al., 2015). They are also capable of maintaining a consistent bioluminescent output intensity under steady state conditions and do not show any toxic effect on the host cell despite the reactive potential of their aldehyde-based luciferin (Close et al., 2011). Rather, unlike the short lived and dynamic bioluminescent signals observed following luciferin supplementation using externally excited systems, the consistent signal of continuously bioluminescent reporters should produce a sufficiently stable signal output to enable reliable detection using less sensitive hardware.

To evaluate the performance of the image intensifier and CMOS-based consumer sensor systems relative to traditional laboratory-based equipment, this work compares their ability to detect and quantify these bioluminescent signals from target cells. These comparisons will provide a basis for better determining if such consumer-focused tools can be used by appropriately trained scientific personnel to enable lower cost, point-of-use human cellular assays and are not to suggest that untrained individuals with access to these systems should endeavor to perform potentially hazardous work. This work compares the detection and performance capabilities of commercially available smartphone (CMOS-based, Google Pixel 4a 5G) and night vision (Image intensifier, AGM Global Vision PVS-12 NL2 night vision monocular) devices with laboratory grade *in vivo* imaging systems (CCD camera-based, IVIS Lumina), and multimode plate readers (PMT-based, BMG CLARIOstar plate reader) for the detection of bioluminescence under common assay objectives.

## Materials and Methods

### Cell Culture and Processing

Continuously bioluminescent LiveLight^TM^ HEK293 cells (490 BioTech) were used for all assays except reporter induction, in which case HEK293 wild type cells (ATCC) were used. All cells were grown in Dulbecco’s Modified Eagle’s Medium (DMEM; Gibco) supplemented with 10% Fetal Bovine Serum (FBS; Gibco), 1× GlutaMAX (Gibco), and 1 × Penicillin/Streptomycin (Gibco). All cultures were maintained at 37°C and 5% CO_2_ in a humidified incubator for routine growth. For CCD-based assays cells were grown in optically opaque black bottom plates. For PMT, image intensifier (night vision), and CMOS (smartphone)-based assays cells were grown in optically opaque white bottom plates.

### Steady State Assays

Minimum signal threshold, linear detection, signal variability, and signal resolution assays were performed using serial dilutions of LiveLight^TM^ HEK293 cells ranging from 2 × 10^5^ to two cells/well in either white or black-walled opaque bottom 96-well plates depending on the imaging method used. Cells were imaged 4 h post plating. This time point was selected because it was sufficient to allow settling and adherence to the plate, but before confluence-based declines in cellular health and growth rate were observed at the highest plating density. Ambient background light levels were measured and maintained as internally consistent for each imaging method and identical between the night vision and CMOS systems.

### Dynamic Signal Assays

For toxicity assays, LiveLight^TM^ HEK293 cells were plated at 2 × 10^4^ cells/well in 96-well plates, incubated overnight at 37°C and 5% CO_2_ in a humidified incubator, and then challenged with either 0, 200, 400, or 800 µg Zeocin (Gibco)/mL. Following challenge, cells were assayed for 24 h and viability was determined relative to the untreated control. For transcriptional induction assays, wild type HEK293 cells were grown in DMEM supplemented with 10% FBS, 1 × GlutaMAX, and 1 × Penicillin/Streptomycin. All cultures were maintained at 37°C and 5% CO_2_ in a humidified incubator for routine growth and transfected with the LiveReport^TM^ CRE (cyclic AMP response element) kit (490 BioTech) according to the manufacturer’s instructions. Twenty-four hours after transfection, cells were challenged with 1 × 10^−5^ M forskolin (Cayman Chemical) and assayed for 24 h.

### CCD Camera Imaging

As a representative CCD-camera-based instrument, bioluminescence was measured using an IVIS Lumina imaging system (PerkinElmer). Luminescent detection was performed across 1 min acquisition periods using medium binning and an F/Stop of 1. A stage temperature of 37°C was used for all assays and measurements were acquired at 1 h intervals.

### PMT Imaging

As a representative PMT-based instrument, bioluminescence was measured using a CLARIOstar multimode plate reader (BMG Labtech). Luminescent detection was performed across 1 s acquisition periods for each well using the automatic gain control settings of the instrument’s firmware. Because this instrument features an atmospherically controlled imaging chamber, cells were maintained at 37°C and 5% CO_2_ throughout the duration of the assay and measurements were acquired at 1 h intervals.

### Image Intensifier Imaging

As a representative image intensifier-based instrument, bioluminescence was detected using a PVS-12 NL2 night vision monocular (AGM Global Vision). Because this instrument does not have innate acquisition capabilities, images were acquired by mounting a camera to the monocular using the supplied adaptor. Assays were performed by maintaining cells at 37°C and 5% CO_2_ in a humidified incubator between readings, then transferring the plate to a dark room under ambient atmospheric conditions for image acquisition at 8 h intervals before returning the cells to the incubator until the next acquisition.

### CMOS Imaging

As a representative CMOS-based sensor, bioluminescence was detected using a Pixel 4a 5G smartphone (Google). The built-in camera on this device has 12.2 MP dual-pixel, 1.4 μm pixel width, and ƒ/1.7 aperture capabilities built upon an IMX363 back-illuminated, stacked CMOS sensor (Sony). Assays were performed by maintaining cells at 37°C and 5% CO_2_ in a humidified incubator until imaging, then transferring the plate to a dark room under ambient atmospheric conditions for image acquisition. To represent usage by an end user with minimal training, photos were taken using the default “Night Sight” settings recommended by the manufacturer for capturing low light images.

### Image Processing Software and Statistical Analysis

Image acquisition and bioluminescence measurements made on the IVIS Lumina instrument were performed using Living Image v4.7.2 Software (PerkinElmer). Bioluminescent measurements made on the CLARIOstar plate reader were processed using MARS Data Analysis Software v3.41 (BMG Labtech). Images obtained using the PVS-12 NL2 night vision monocular and Pixel 4a 5G smartphone were processed using ImageJ v1.52 (National Institutes of Health). All statistical analyses were performed using Microsoft Excel. Average values were calculated as arithmetic means. Errors were calculated as ± the standard error of the mean (S.E.M.) for all measurements. Statistical differences between groups were identified using Student’s t-tests with *p*-value cut offs of *p* ≤ 0.05. Pearson’s correlation coefficients were used to determine R^2^ values showing relationships between different parameters.

## Results

### Ambient Light Measurement and Standardization for Comparison Between Systems

Ambient light detection was measured for each of the assay systems. The plate reading chamber within the PMT-based plate reader had the lowest level of ambient light at 3.12 × 10^4^ (±6.15 × 10^3^) photons/sec. The imaging chamber of the CCD camera-based system was the second lowest, with a measured value of 3.04 × 10^5^ (±7.45 × 10^4^) photons/sec. The image intensifier and CMOS systems were both tested in the same location under low light conditions. The background for those measurements was slightly higher (2.88 × 10^6^ (±7.13 × 10^5^) and 2.65 × 10^6^ (±6.63 × 10^5^), respectively). However, these values were not statistically different from one another (*p* = 0.8249). To enable comparison between the systems that report values in arbitrary units (PMT, image intensifier, and CMOS), a standard was measured in each device and a conversion factor was determined to convert values to photons/sec. Each RLU reported by the PMT corresponded to 45.7 photons/sec. Each raw integrated density reported by the image intensifier corresponded to 64.5 photons/sec. Each raw integrated density reported by the CMOS corresponded to 277.8 photons/sec.

### Minimum Signal Detection Threshold

**FIGURE 1:**
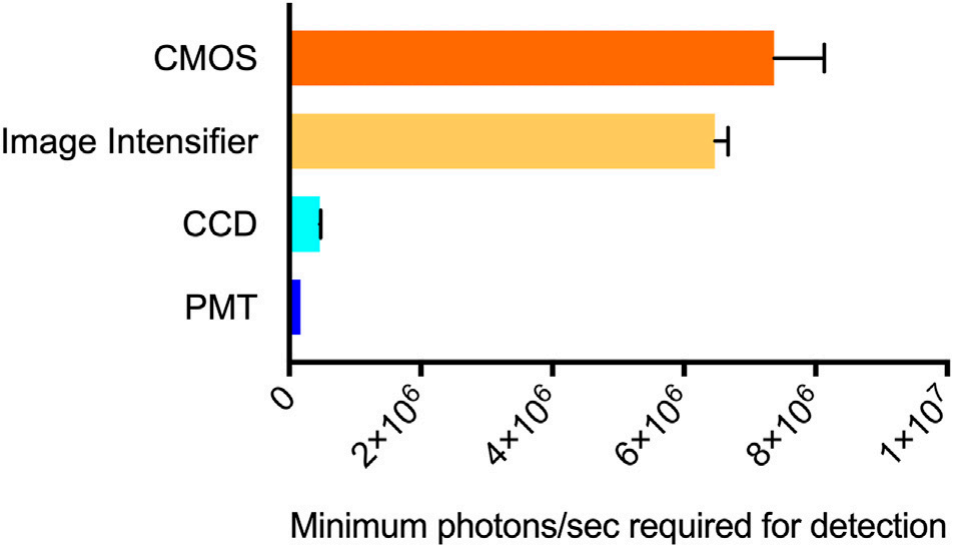
Minimum signal detection threshold for each system. The CMOS (smartphone) system required the highest level of flux to detect bioluminescence at 7.37 × 10^6^ photons/sec. The image intensifier (night vision) system was slightly more sensitive, requiring a minimum of 6.47 × 10^6^ photons/sec. The CCD and PMT systems had lower minimum detection thresholds of 4.65 × 10^5^ and 1.7 × 10^5^ photons/sec, respectively. *n* = 3; error = S.E.M.

CCD camera-based imaging detected bioluminescence down to a minimum threshold of 200 cells/well. Based on the photon counts from the CCD camera, this represents a flux requirement of 4.65 × 10^5^ (±1.27 × 10^4^) photons/sec. PMT-based imaging detected luminescence at a minimum threshold of 20 cells/well. This represents a minimum flux requirement of 1.7 × 10^5^ (±2.26 × 10^4^) photons/sec. The image intensifier and CMOS-based systems were less sensitive. Both displayed a minimum signal detection threshold of 2 × 10^3^ cells/well. Their minimum flux requirements were measured as 6.47 × 10^6^ (±1.98 × 10^5^) photons/sec (image intensifier) and 7.37 × 10^6^ (±7.63 × 10^5^) photons/sec (CMOS) (Figure 1). All systems successfully detected bioluminescence at cell counts representative of common cellular plating densities for assays performed in 96-well plates (ATCC, 2021) and at flux values below what could be detected by the naked eye.

### Linear Detection Capabilities

**FIGURE 2:**
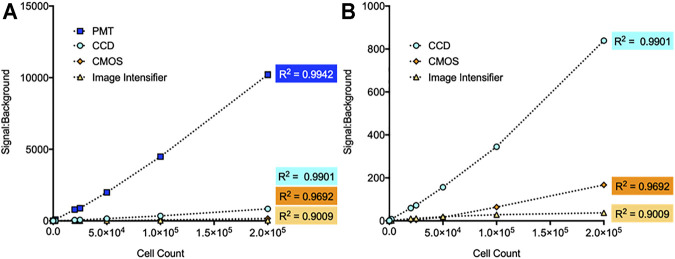
Comparison of linear detection capabilities for different imaging systems. **(A)** Each system showed a high correlation between measured signal:background and cell count. The PMT system had the strongest correlation (R^2^ = 0.9942), followed by the CCD system (R^2^ = 0.9901), CMOS system (R^2^ = 0.9692), and image intensifier system (R^2^ = 0.9009). **(B)** Zoomed view of the CCD, CMOS, and image intensifier data points. *n* = 3; error = S.E.M.

The linear detection capabilities of each imaging method were determined by serially diluting continuously bioluminescent LiveLight^TM^ HEK293 cells (490 BioTech) from 2 × 10^5^ to two cells/well to ensure measurement across the full spectrum of detectable cell numbers for each system as determined above. The minimum detection threshold of each system observed during this experiment remained consistent with the previous values. Both the CCD and PMT-based imaging systems showed strong linear correlations between the plated cell number and measured bioluminescent signal (R^2^ = 0.9901 and 0.9942, respectively). CMOS-based imaging also had a strong linear correlation (R^2^ = 0.9692). The image intensifier-based system was highly correlated (R^2^ = 0.9009), but less capable of identifying a linear correlation compared to other imaging methods (Figure 2). This may be indicative of a lower signal:background ratio resulting from performance of the measurement outside of a light tight imaging chamber, or it may be due to higher detection variability. The latter possibility is further explored below.

### Variability of Steady State Signal Detection

**FIGURE 3:**
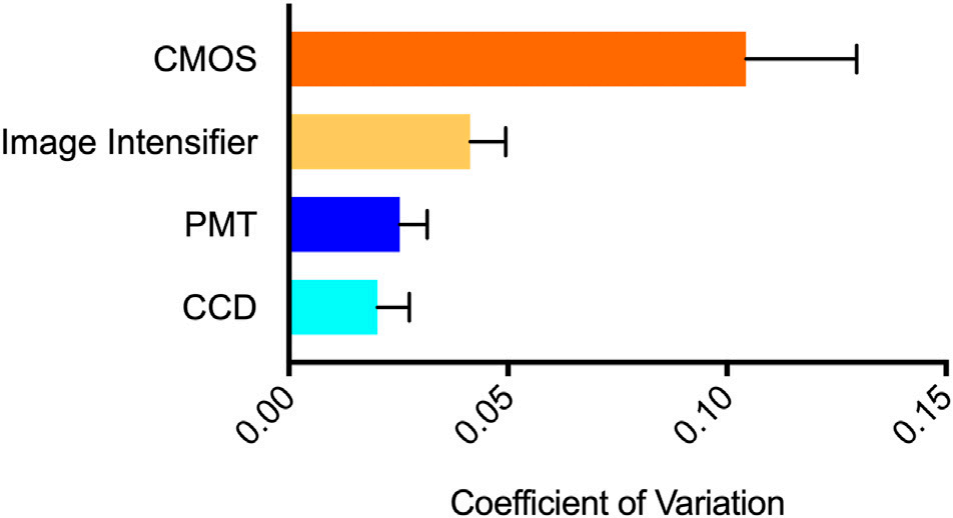
Variability of steady state signal detection visualized using coefficient of variation. The CMOS system showed the highest variability (CV = 0.1043), followed by the image intensifier system (0.0414), PMT system (0.0252), and CCD system (0.0202). *n* = 6; error = S.E.M.

The coefficient of variation (CV) was calculated for each system to determine its variability during steady state signal acquisition (Figure 3). Individual CV values were obtained from replicate measurements made using six different concentrations of cells to determine if signal intensity affected variability. However, no intensity-based effects were observed. The CCD, PMT, and image intensifier devices had statistically similar (*p* = 0.1477) CV values of 0.0202, 0.0252, and 0.0414, respectively. The CMOS device had a CV of 0.1043, which was statistically different than the other three imaging methods (*p* = 0.0394).

### Signal Resolution Limitation

**FIGURE 4:**
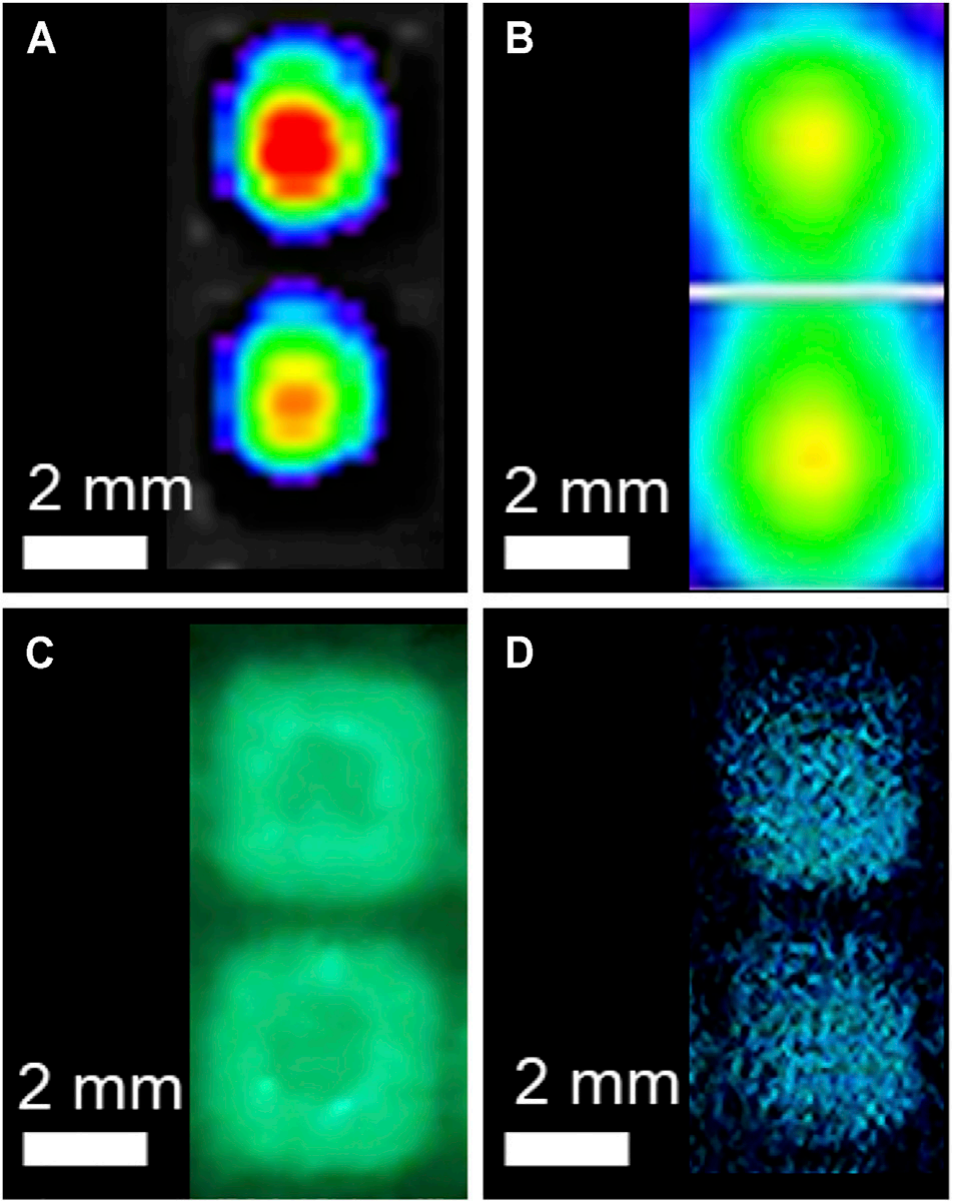
Signal resolution testing of the CCD, PMT, image intensifier, and CMOS detection systems. The **(A)** CCD, **(C)** image intensifier, and **(D)** CMOS systems all successfully resolved individual signals separated by 0.87 mm following plating of continuously bioluminescent cells in the 384-well plate format. The **(A)** CCD and **(B)** PMT images are pseudocolor images with warmer colors representing relatively increased signal intensity. The **(B)** PMT-based plate reader was not able to acquire signal from multiple wells simultaneously, so each pixel in this image represents a 1.1 μm^2^ area within the well to demonstrate the resolution of signal discrimination within the well.

To determine each systems ability to discriminate spatially constrained independent signals, LiveLight^TM^ HEK293 cells were plated in the 384-well format. This created a 0.87 mm non-luminescent separation between wells. The CCD, image intensifier, and CMOS systems all successfully resolved individual wells at this distance (Figure 4). The PMT-based image, which was obtained in a plate reader and therefore not capable of observing multiple wells simultaneously, was tested by performing a 30 × 30 matrix well scan to generate a pseudocolor image representative of bioluminescence intensity within the well at a resolution of 1.1 μm^2^/pixel. Both the CCD and PMT produced pseudocolor images based on luminescent intensity. The image intensifier and CMOS images could be directly observed.

### Detection of Toxicity

**FIGURE 5:**
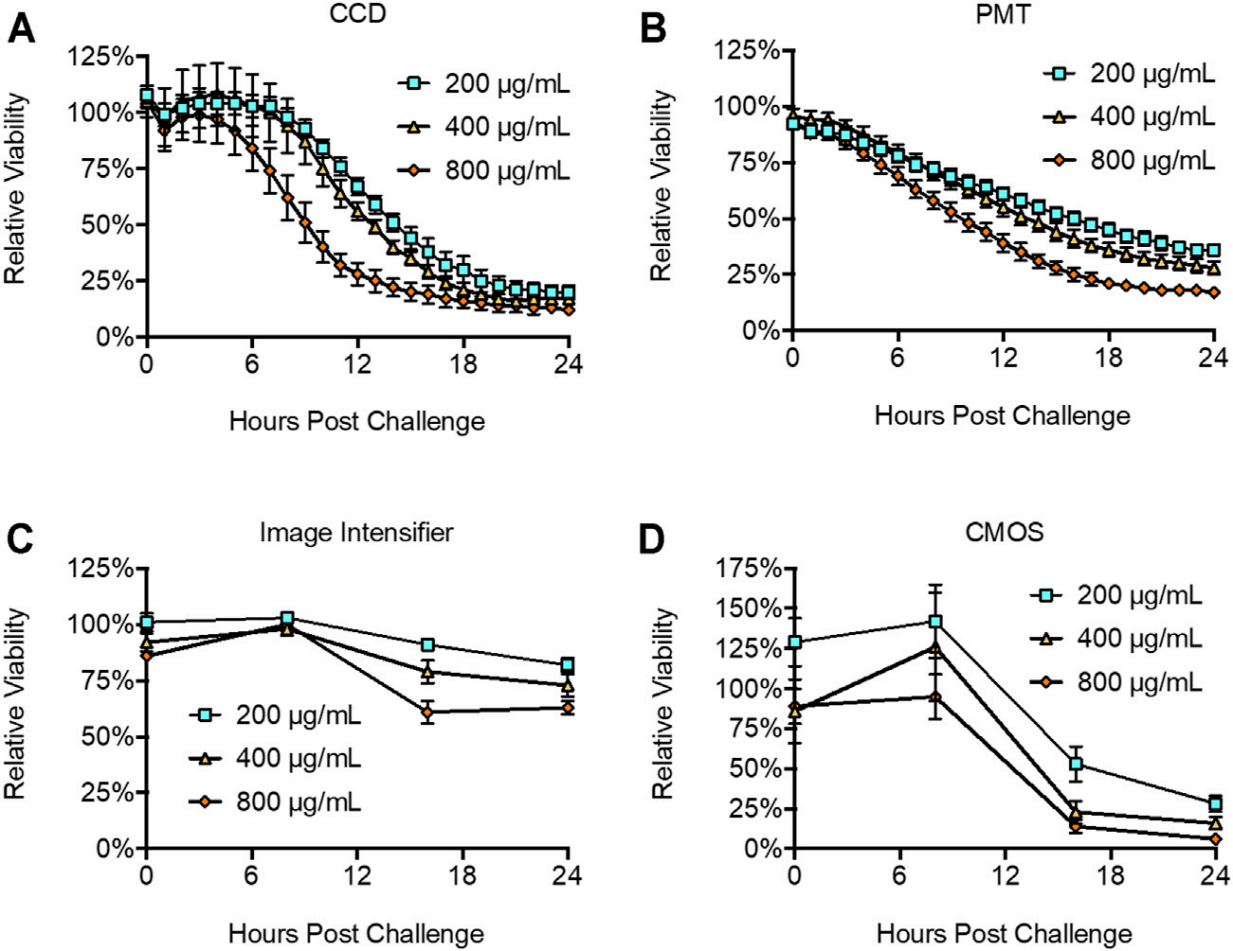
Detection of toxicity following Zeocin challenge. The **(A)** CDD, **(B)**, PMT, **(C)** image intensifier, and **(D)** CMOS systems all successfully reported declines in cellular health following Zeocin challenge. The **(B)** PMT system was the most sensitive, reporting significant differences at 1 h post challenge. The **(A)** CCD system did not discriminate a statistically significant change until 8 h post challenge, while the **(C)** image intensifier and **(D)** CMOS systems both required 16 h to identify a statistically significant change. Untreated control is defined as 100% viability. *n* = 3; error = S.E.M.

Continuously bioluminescent LiveLight^TM^ HEK293 cells were plated at 2 × 10^4^ cells/well in a 96-well plate and challenged with increasing concentrations of the cytotoxic chemical Zeocin to monitor toxicity. Zeocin induces toxicity by intercalating into DNA and inducing double strand breaks that ultimately compromise genomic integrity and result in cell death (Ehrenfeld et al., 1987). CCD imaging identified a significant (*p* = 0.0468) decrease in cellular health at 8 h post treatment from the 800 μg/ml challenge, with toxicity decreasing in a dose dependent pattern for lower challenges (Figure 5A). PMT-based imaging identified a statistically significant (*p* = 0.0268) decrease in cellular health within 1 h post treatment at the 800 μg/ml challenge. It also showed toxicity decreasing in a dose dependent manner and detected statistically significant decreases from all challenge levels after the 3 h time point (200 μg/ml, *p* = 0.0104; 400 μg/ml, *p* = 0.0095; 800 μg/ml, *p* = 0.0028) (Figure 5B). The image intensifier and CMOS-based systems identified statistically significant changes in cellular health at the 800 μg/ml (*p* = 0.0003 and 0.0023), 400 μg/ml (*p* = 0.0063 and 0.0041), and 200 μg/ml (*p* = 0.0062 and 0.0075) dosage levels in a dose dependent manner (Figures 5C,D). However, this detection was not observed until 16 h post challenge.

### Detection of Reporter Induction

**FIGURE 6:**
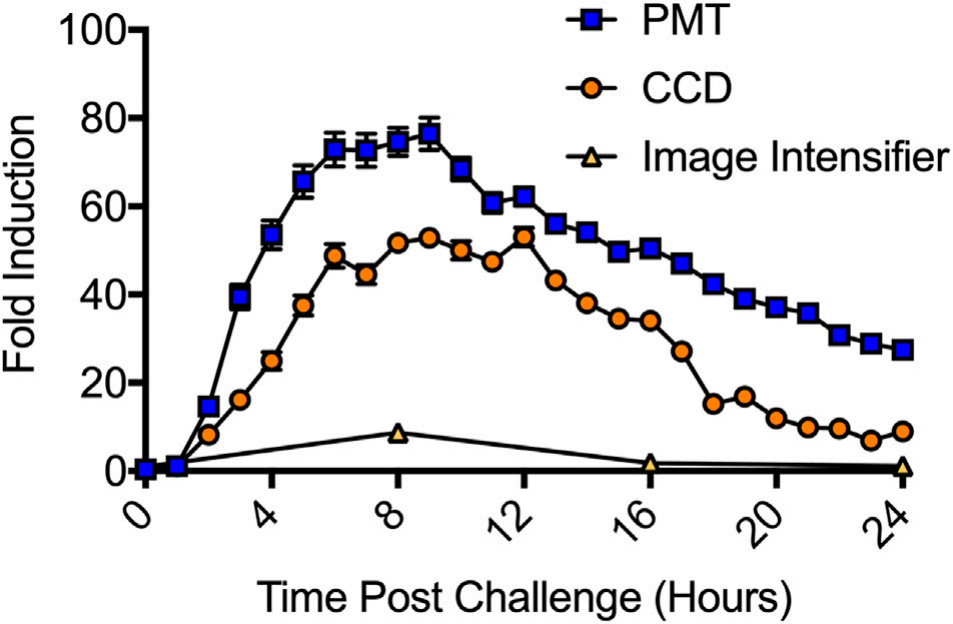
Detection of CRE induction following forskolin treatment. The PMT system was the most sensitive system tested. It detected significant induction at 2 h post treatment and recorded a maximum induction of 76-fold. The CCD system was the second most sensitive. It also detected significant induction at 2 h post treatment, but only recorded a 53-fold maximum induction. The image intensifier system required 8 h to report a significant induction and only observed a 9-fold maximum induction. The CMOS system (not shown) did not detect significant induction at any surveyed time point. *n* = 3; error = S.E.M.

The LiveReport^TM^ CRE Assay Kit (490 BioTech) was used to compare each systems’ ability to detect bioluminescent reporter induction. The LiveReport^TM^ CRE assay kit was transfected into wild-type HEK293 cells according to the manufacturer’s instructions. Transfected cells were treated with forskolin to activate cyclic AMP response element (CRE) expression and assayed over a 24 h period. CCD imaging detected significant (*p* = 0.0074) induction beginning at 2 h post treatment, with peak induction ranging from 6 to 12 h post treatment and maximum induction reaching 53-fold over control (Figure 6). PMT imaging detected significant (*p* = 0.0005) induction starting 2 h post treatment, with peak induction ranging from 5 to 10 h post treatment and a maximum induction of 76-fold over control. For both CCD and PMT imaging, induction was still significant (*p* = 0.0001 for both) 24 h post treatment. The image intensifier identified peak induction at 8 h post treatment, with a 9-fold increase over control. Induction was still statistically significant (*p* = 0.0001) at 16 h post treatment, but was no longer significant (*p* = 0.0893) at 24 h post treatment. CMOS imaging did not detect induction.

## Discussion

This study compares how specialized, laboratory-focused bioluminescent imaging systems (CCD camera-based *in vivo* imaging system and PMT-based multi-mode plate reader) perform relative to less expensive, more mobile, consumer-focused alternatives (image intensifier-based night vision and CMOS-based smartphone camera). However, it is important to note there is a wide range of CCD, PMT, image intensifier, and CMOS-based systems available, and this study only explores one example of each. Each imaging method is also dependent on the software used for image processing. In this regard, the laboratory focused IVIS Lumina and BMG CLARIOstar both use proprietary software that is provided with the instruments at the time of purchase. These software packages are responsible for both data processing and machine management. It is therefore likely the results obtained in these tests would be different if alternative software was used to control machine functionality and collect raw data from the instrument sensors. Because they are not designed primarily for laboratory usage, the AGM PVS-12 NL2 monocular and Google Pixel 4a 5G do not come with software specifically for this task. Therefore, we obtained image files from these instruments and analyzed them with the open source ImageJ software, which is commonly utilized for such applications and widely available. Just as with the laboratory-focused instruments, it is likely that use of an alternative image processing software could provide varied results. Furthermore, especially in the consumer-focused smartphone market, functionality improvements are occurring at a rapid pace and improved hardware will likely become available at similar price points in the near future. Therefore, the use of alternative equipment may have a significant impact on the results presented here.

Using the continuously bioluminescent signal of LiveLight^TM^ HEK293 cells, both the night vision and smartphone systems could detect bioluminescence from as few as 2,000 cells/well. CCD imaging could detect bioluminescence from 200 cells/well, and PMT imaging could detect down to 20 cells/well (Figure 1). As expected, these data show that the CCD and PMT systems, which are designed for laboratory use, are more sensitive and have improved signal:background ratios compared to the consumer-grade alternatives. However, both night vision and smartphone-based imaging could detect bioluminescence from well below the range of common densities used for assays run in the 96-well plate format (ATCC, 2021). The CCD and PMT-based systems both showed very strong linear correlations between bioluminescence and cell number, with each achieving R^2^ values ≥0.99. However, the night vision and smartphone systems were only slightly less correlated, with both achieving R^2^ values ≥0.9 (Figure 2).

All surveyed systems showed CV values ≤0.1 (Figure 3), with the night vision system performing relatively similar to the CCD and PMT systems despite necessitating use outside of a light tight environment. The practical effect of the measured CV values was reflected when measuring viability. The CCD (Figure 5A) and PMT-based (Figure 5B) systems, which had the lowest CV values (Figure 3), show clear downward trends in viability after compound challenge. In contrast, the CMOS-based system (Figure 5D) appears to indicate an unexpected increase in viability between chemical challenge and the 8 h time point, followed by a significant decrease towards the 16 h time point. It is likely this reported increase, which was not statistically significant (*p* = 0.52), is an artifact of the higher CV associated with this modality and the minor difference between the time zero and 8 h points (86 and 126%, respectively) are due to chance. A similar effect was observed in the night vision system (Figure 5C), which appears to show slight increases in viability between time zero and the 8 h time point for the 400 and 800 μg Zeocin/mL treatments. Just as this modality displayed a CV between the lower variability CCD and PMT-based systems and the more variable CMOS-based system (Figure 3), it also showed a less pronounced difference between the suspect data points and relatively lower *p* values (0.0468 and 0.0007) for the two treatments than was observed for the CMOS-based system at that same time point. For all systems, variation remained consistent across a large range of bioluminescent intensities.

The sensitivity of these systems was highlighted by their performance in the toxicity and reporter induction assays. PMT-based imaging detected cellular health dynamics as early as 1 h post treatment, while CCD-based imaging required 8 h. The night vision and smartphone-based systems both required 16 h to observe a significant change (Figure 5). This can be attributed at least partially to the logistical constraints of these systems. While the CCD and PMT systems could be programmed to continuously collect data every hour, the night vision and smartphone systems were limited by their requirements for manual operation and inability to maintain the cells under sterile, atmospherically favorable conditions during imaging. As such, they were only assayed at 8 h intervals with the cells housed in an incubator between readings, instead of every 1 h like the CCD and PMT systems. Therefore, although the first detectable difference was recorded at 16 h post treatment, it is likely they could achieve detection earlier if an alternative imaging schedule was used. Overall, the PMT-based system displayed the highest sensitivity and signal:background ratio, with the CCD-based system only slightly less sensitive. Interestingly, the night vision and smartphone-based systems performed roughly similarly, despite their very different photon detection methodologies. All systems could detect Zeocin-based toxicity within the manufacturer’s suggested range of 50–1,000 μg/ml. However, only the more sensitive CCD and PMT systems showed the two treatments within the average selective range of 200–400 μg/ml as being similar to one another while remaining distinct from the higher 800 μg/ml dosage that is more concentrated and therefore more likely to show toxic effects earlier during the selection process (ThermoFisher, 2021).

The reporter induction assay (Figure 6) was a more difficult challenge because it yielded lower overall signal intensities than the toxicity assay. Both the CCD and PMT systems detected significant induction (*p* < 0.01) at the 2 h time point and reported maximal induction values of 53-fold and 76-fold, respectively. The night vision system also successfully identified induction and reported a maximal induction of 9-fold. However, it was again limited by a requirement for manual operation and inability to maintain the cells under sterile, atmospherically favorable conditions during imaging. While it detected significant (*p* = 0.0001) induction at the first possible time point (8 h), and while both the CCD and PMT systems still showed induction at this time, it suffered from a significant reduction in data resolution compared to these other assays. Although the CCD, PMT, and night vision systems all showed roughly the same timing for the start (2 h) and peak (8 h) of induction, they produced disparate maximal induction values. These values correlated with the measured background levels, sensitivities (Figure 1) and linear detection capabilities (Figure 2) of each system. It is therefore likely the discrepancies in reported maximal induction are due to the ability of each system to discriminate signal from background. Those with improved signal:background discrimination abilities, resulting from both their improved signal detection capabilities and improved ambient light exclusion properties (PMT and CDD), report higher maximal induction values. The image intensifier system, which displayed a relatively decreased discrimination ability and greater ambient light exposure, reported a lower maximal induction because it could not as easily distinguish induction from background.

Smartphone-based imaging did not detect reporter induction. However, the results of the minimum signal threshold testing (Figure 1) suggest the LiveReport™ CRE induction signal was only slightly below the luminescent threshold required. The Pixel 4a 5G CMOS sensor used in these experiments has a 1.4 μm pixel size. This was sufficient for resolving the spatial location of disparate signals down to the 384-well format. However, using a sensor with a larger pixel size could potentially increase photon detection capabilities by allowing more light to be captured by each pixel and increasing minimum signal detection capabilities. Since most assays are run in 96-well formats that have greater spacing between wells, this approach would tolerate the decreased resolution of larger pixels while increasing sensitivity, which was determined to be the most limiting factor for this sensor type. Although smartphone photography often emphasizes increased resolution, and therefore future versions of these devices are likely to incorporate CMOS sensors with decreased rather than increased pixel size, technical improvements in the CMOS space have focused on improving dark count rates and photon detection probabilities (Charbon et al., 2018). Given the performance of modern back-illuminated, stacked CMOS sensors, and the focus smartphone manufactures have placed on photographic quality improvement across a range of metrics, it is therefore likely an alternative camera system, or a near-future smartphone camera would be successful in this assay.

CCD and PMT-based instruments are the primary tools for bioluminescent assays in laboratories around the world because of their high sensitivity and the decision to use one over the other is typically application dependent (de la Zerda et al., 2010; Kim et al., 2020). However, while their relatively large footprint, high price, immobility, and resource requirements limit their use outside of the laboratory environment, ongoing miniaturization of PMTs is improving their ability for portable usage. The rapid improvement in night vision and especially CMOS-based smartphone imaging, along with complementary improvements in bioluminescent technologies that allow for continuous signal generation and autonomous signal control without external activation, has opened new possibilities for performing assays without these specialized instruments. When paired with autonomously bioluminescent cells, night vision and smartphone imaging can now provide an inexpensive, compact, and highly mobile alternative for bioluminescent imaging if working with applications capable of producing signal above their higher minimum detection thresholds. As these devices improve they could be used to enable a wide range of novel applications that span from performing point-of-care toxicity assays in remote locations to providing educational experiments in classrooms at all ages.

However, it is unlikely that night vision or smartphones will replace CCD and PMT-based systems in scientific laboratories anytime soon. Aside from their improved performance as showcased in this work, these systems are typically integrated with temperature control, atmospheric control, wavelength filtering, continuous integration, and a host of other accessories that allow them to perform myriad functions currently inaccessible to consumer-focused products. As retailed, night vision and smartphone systems lack these abilities. However, it is encouraging that with only minor modifications (i.e., addition of an external filter to reduce background and improve signal:background ratio) they can be employed to perform similar assays in situations inaccessible to laboratory-based systems.

## Data Availability

The raw data supporting the conclusions of this article will be made available by the authors, without undue reservation.
